# Toward a Green and Sustainable Silver Conservation: Development and Validation of Chitosan-Based Protective Coatings

**DOI:** 10.3390/ijms232214454

**Published:** 2022-11-21

**Authors:** Francesca Boccaccini, Chiara Giuliani, Marianna Pascucci, Cristina Riccucci, Elena Messina, Maria Paola Staccioli, Gabriel Maria Ingo, Gabriella Di Carlo

**Affiliations:** 1 Institute for the Study of Nanostructured Materials (ISMN), National Research Council (CNR), Via Salaria km 29300, 00015 Monterotondo, Italy; 2 Department of Earth Sciences, Sapienza University of Rome, Piazzale Aldo Moro, 5, 00185 Rome, Italy; 3 Division Sustainable Materials, Italian National Agency for New Technologies, Energy and Sustainable Economic Development (ENEA), C.R. Casaccia, Via Anguillarese 301, S. M. Di Galeria, 00123 Rome, Italy

**Keywords:** green polymers, silver alloy, chitosan coatings, corrosion inhibition, artifacts conservation

## Abstract

When exposed to air, silver artifacts undergo an unpleasant darkening and shiny loss, commonly known as tarnishing. At the present, the development of protective coatings by using eco-friendly and biocompatible materials, able to ensure high transparency and to hinder the degradation of silver objects, remains a huge challenge. In this study, chitosan was used for the first time to realize sustainable coatings for silver protection. Both pure and benzotriazole-containing chitosan coatings were prepared and applied on sterling silver disks. A commercial product based on acrylic resin was used as a reference. The aesthetic features and protective properties of these coatings were evaluated by performing two different types of aging treatments. In particular, the assessment of the protective efficacy was carried out by reproducing both highly aggressive polluted environments and real-like museums’ storage conditions. In the first case, chitosan-based coatings with benzotriazole performed better, whereas in storage conditions all the chitosan films showed comparable efficacy. Compositional, morphological and structural analyses were used to evaluate the protective properties of the coatings and to detect any physical or chemical modifications after the aging treatments. Our findings reveal that the two different testing methods provide complementary information. Moreover, chitosan coatings can achieve protective efficacy comparable with that of the commercial product but using non-toxic solvents and a renewable biopolymer. Chitosan coatings, designed for cultural heritage conservation, are thus promising for the protection of common sterling silver objects.

## 1. Introduction

While the research on the corrosion protection of Ag-based components to be used in electronic devices has developed many valuable and highly efficient solutions [1], the effective and sustainable protection of Ag-based artifacts is still a challenge for scientists and conservators. Due to interactions with environmental aggressive species, such as those containing sulfur and chlorine, silver surfaces quickly acquire an unpleasant dark gray color, losing their characteristic brightness and shine [2,3]. This process, commonly known as tarnishing, is the result of a series of electrochemical reactions, involving both metal elements and atmospheric gaseous species, and it is promoted by high values of relative humidity [4]. Copper is usually added as an alloying element to pure silver, with the aim of improving hardness and strength [5]. Due to the scarce solid miscibility of Ag and Cu, this latter crystallizes in the form of dispersions among a silver-enriched matrix, resulting in a heterogeneous and highly reactive microstructure [6]. Galvanic coupling between nobler Ag-enriched regions (β-phase) and less noble Cu dispersions (α-phase) leads indeed to the establishment of cathodic and anodic areas, respectively, where corrosion processes can start and proceed. As a result, a mixture of silver and copper compounds (especially oxides and sulfides), which has developed on the artifact’s surface, is responsible for the unpleasant darkening [3].

In order to protect silver objects from tarnishing, protective coatings are typically applied on their surface [7,8]. The most used materials are based on organic resins, such as acrylic ones, which are dissolved in a mixture of organic solvents and which usually incorporate corrosion inhibitors, such as benzotriazole (BTA) [9]. These formulations, in addition to protective properties, have to fulfill a series of aesthetic requirements and to be completely removable. They must be transparent, colorless and maintain such features for as long as possible. Unfortunately, commonly used protective coatings, such as those based on acrylic resins, suffer some drawbacks. They can undergo physico-chemical modifications that, in the long-term, lead to the film’s yellowing and embrittlement, along with difficulties with the coating removal. Moreover, they contain corrosion inhibitors and are applied and removed using organic solvents (e.g., toluene, xylene, acetone) that are harmful for both conservators and the environment [10].

In this scenario, a very interesting green polymer with a remarkable film-forming ability and biocompatibility is chitosan. It is an organic copolymer composed by repetition of D-glucosamine and N-acetyl-glucosamine units, linked by β-(1-4) covalent bonds and obtained by the partial deacetylation of chitin, one of the most abundant polymers found in nature and industrially extracted from crustaceans’ shells [11]. It can be dissolved in water, thus avoiding the use of harmful solvents, even if a slightly acidic pH has to be achieved. Due to its several advantageous properties, chitosan has received interest for applications in many fields, including biomedicine, cosmetics, environmental recovery and textile industry [12,13]. The biocompatibility and the film-forming properties make chitosan a suitable candidate to develop protective coatings for metal substrates and also a possible alternative to the toxic commercial products that are currently used by conservators. In the field of metal protection, it has been proposed as a coating for industrial steel, aluminum and copper [14,15,16,17,18], while a recent work, developed in the frame of the European-funded project NANORESTART [19], has focused on the formulation and application of chitosan-based coatings on a modern bronze alloy [20,21]. To the best of our knowledge, no published works report about the use of chitosan or other biocompatible polymers for the protection of Ag-based substrates. Therefore, efforts are still necessary for an effective and sustainable conservation of silver artifacts.

In this study, we propose chitosan as a promising candidate for the sustainable protection of modern Ag-based artifacts. The aim was to test the coatings previously developed for bronze alloys on sterling silver substrates to be preserved in indoor environments [20,21]. Two formulations, a pure chitosan and a benzotriazole-containing one, were chosen and studied in comparison to Incral 44, a commercial acrylic varnish commonly used for the protection of silver artifacts. To validate the coatings, a proper reference alloy, reproducing both chemical composition and microstructure of as-cast Ag-based artifacts, was produced [22], coated with the chitosan-based films and with the commercial reference and used to perform artificial aging treatments. These were developed in order to reproduce two different environments: a highly aggressive atmosphere enriched in sulfide ions and a real-like polluted context, typical of unsuitable museums’ showcases or storage boxes. It was then possible to monitor, respectively, short-term and long-term coatings behavior, as well as to evaluate their protective efficacy in different conditions. Moreover, polymer/metal surface interactions were examined and further investigated, as well as coatings’ degradation processes. A set of analytical techniques was used in this study. Optical microscopy (OM) was used to visually evaluate the disks’ surface modifications after the tests, while field emission scanning electron microscopy (FE-SEM) coupled with energy dispersive spectrometry (EDS) was used to study morphological and compositional modifications at the nano- and microlevels. UV-Vis spectroscopy was employed to study the coating transparency and the formation of degradation products. Fourier transform infrared spectroscopy (FTIR) was used in combination with FE-SEM-EDS analysis to study the coatings removability. X-ray diffraction (XRD) was also used to characterize the corrosion products grown on the reference alloy surface after the aging treatments.

## 2. Results and Discussion

In this study, two different types of chitosan-based coatings were considered: pure chitosan films and chitosan films with benzotriazole. The first coatings were studied with the aim of evaluating the protective properties of pure chitosan toward sterling silver alloy. The numerous hydroxyl and amino functional groups of the polymer can play a role in the stabilization of the metal surface, since they can act as sites for coordination bonds. Previous studies have indeed demonstrated how hydroxyl and amine functions in chitosan are directly involved in the uptake of metal cations through chelation mechanisms [23], resulting in the formation of strong polymer/metal interactions, especially in the case of copper ions [24]. Moreover, chitosan is able to chemically bond copper surfaces, leading to the formation of a well adherent and chemisorbed polymeric layer onto the metal substrate [16]. Chitosan-based films can thus potentially be effective in the corrosion protection of silver alloys, providing surface passivation through physico-chemical interactions. They can also form a barrier able to limit the contact between the underlying metal and external aggressive species. In order to enhance the protection properties, the addition of a corrosion inhibitor was decided. Although a recent work reported that benzotriazole’s protective efficacy on silver surfaces is unsatisfactory [25], at the present BTA it is still the most appreciated inhibitor used to passivate Ag-based alloys, and for this reason it was selected in our study [26]. When dealing with corrosion inhibitors, and in particular benzotriazole, a tricky issue is the environmental and health risk. Even though the toxicity of BTA and its derivatives toward microorganisms, plants and invertebrates was deeply investigated and ascertained [27], studies about adverse effects on human health are still a few [28]. In any case, a small percentage of BTA was used with respect to the polymer for the formulation of our coatings. Moreover, the incorporation inside a polymeric matrix strongly reduces contact and leaching into the environment.

### 2.1. Aesthetic Properties and Removability

**Figure 1:**
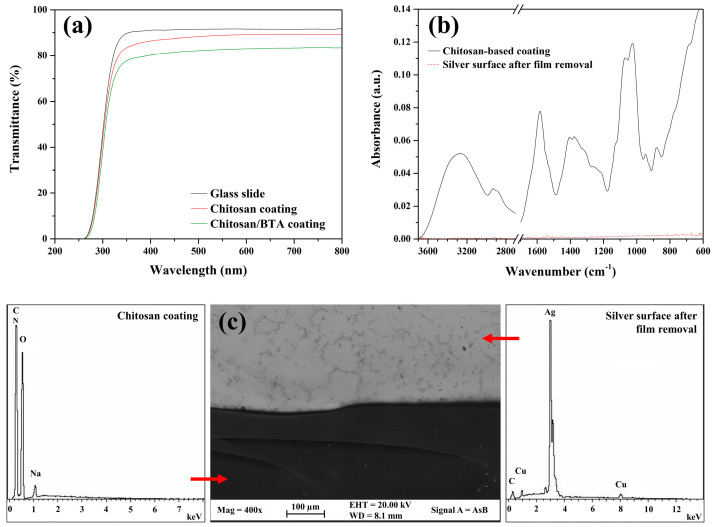
Transparency and removability of chitosan-based coatings. (**a**) UV-Vis transmittance spectra of a glass slide and of chitosan and chitosan/BTA coatings applied on the same glass slide; (**b**) ATR-FTIR spectra acquired on the chitosan coating and on the silver surface after film removal; (**c**) FE-SEM-EDS analyses on the chitosan coating and on the underlying Ag alloy after film removal.

Protective coatings applied on artistic objects have to fulfill particular requirements. They must be transparent, colorless and easily removable. UV-Vis spectra, collected on both coatings’ typologies applied on a glass slide, resulted to be very similar to the spectrum of the glass slide alone (Figure 1). The high values of transmittance and the flat trend in the region of visible light (380–780 nm) confirm that chitosan-based coatings totally fulfill transparency and absence of color, respectively. Moreover, they are completely water soluble and totally biocompatible. These features represent a very important property for an easy and safe application by conservators. They can be applied on silver substrates (e.g., by brushing or spraying) and can be easily removed at any time without the need for chemical hoods or protective devices. When the need of coating removal arises in fact, a simple gauze, soaked in water or ethanol, is enough to take the coating off, without leaving any visible residues of the film on the silver surface. In addition, chitosan-based coatings applied on silver artifacts can be touched and handled without any risks of the film’s failure. Figure 1b,c show, respectively, the results of the ATR-FTIR and FE-SEM-EDS analyses acquired on the coated silver surface and on the uncoated one after the film was removed. The coating’s residues cannot be detected, thus confirming the reversibility of the chitosan-based films.

### 2.2. Accelerated Corrosion Test

Most of the studies about tarnishing phenomenon and anti-corrosion materials are conducted subjecting samples to artificial aging treatments by immersion in aggressive solutions [29,30,31]. This highly corrosive environment may be not well representative of the real conditions to which artifacts are currently exposed inside museums or storage cases [2,32]. The mechanism and rate of the reactions can indeed vary depending on whether the surrounding media is liquid or gaseous. Moreover, the water solubility of the chitosan-based coatings needs a different procedure. For these reasons, it was decided to expose the metal disks, both bare and coated, to sulfur vapors in a closed vessel. After some tests using different ammonium sulfide concentrations, the 1 mM was selected as the right one. On the one hand in fact, this concentration proved to be the best choice to induce the deterioration of silver surfaces in short times, while on the other hand it allowed us to appreciate any differences in treatment response between bare and protected disks.

The surface degradation induced by this accelerated artificial aging resulted in agreement with the tarnishing effects observed on real artifacts. The XRD pattern (Figure S1) recorded after 36 h of exposure of a bare alloy to high-concentrated ammonium sulfide vapors, revealed the presence of copper hydroxide and silver and silver-copper sulfides (namely acanthite, Ag_2_S, and jalpaite, Ag_3_CuS_2_). These alteration compounds, especially the sulfides, are commonly detected on silver artifacts that have suffered tarnishing phenomenon in indoor polluted environments [2,32].

**Figure 2:**
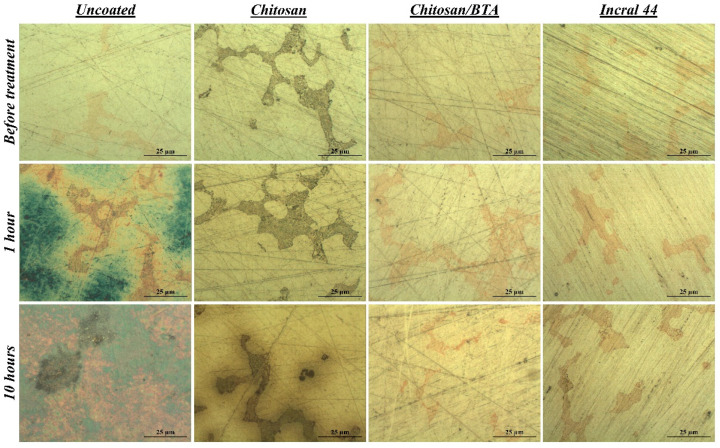
Surface features before and after the accelerated corrosion test. Optical microscopy images of a bare, a chitosan, a chitosan/BTA and an Incralac 44 coated disk at different times of artificial aging treatment.

The protective efficacy of chitosan-based coatings was firstly evaluated by comparing surface modifications occurring at the bare and coated metal surfaces after the exposure to the accelerated aging treatment. The examination was carried out by means of an optical microscope enabling the direct visualization of the underlying metal surface, without removing the superimposed protective film. In Figure 2, optical microscopy images of the disks’ surfaces, both bare and coated with chitosan, chitosan/BTA and Incral 44 films, are reported for different times of aging. Before the treatment, the surface of every sample appeared smooth, clean and characterized by two coexisting phases: a silver-rich matrix and embedded copper-rich orange dispersions. Such heterogeneity, invisible to the naked eye, makes this alloy highly reactive and explains the characteristic alteration mechanism observed on the bare surfaces (Figure S2). In regard to the sample covered with the pure chitosan coating, the copper-rich areas appeared locally darkened, probably due to a slight oxidation process promoted by the chitosan solution during application. In the case of chitosan/BTA, this was not observed. It is well known, in fact, that benzotriazole acts as an excellent corrosion inhibitor for copper, preserving it from oxidation and corrosion [33]. After one hour of treatment, the differences between the coated and uncoated surfaces were very pronounced. The disks covered with the protective coatings did not change, whereas the bare disk underwent deep color modifications attributable to the growth of corrosion products on its surface. Carrying out the treatment up to ten hours, some differences between chitosan and chitosan/BTA-coated surfaces started to be visually detectable. Concerning the disk covered with the pure chitosan film, a brown halo formed along the copper dispersions, which in turn became darker. On the contrary, the disk covered by chitosan/BTA did not undergo noticeable changes and its surfaces remained cleaned such as those covered by the commercial reference coating (Figure S3). This suggests that the coating containing the inhibitor ensured a better protective action especially toward the copper-rich areas. Nevertheless, the surface modifications induced by the sulfur vapors were far less pronounced on the samples protected by the chitosan-based coatings, especially if compared with the ones suffered by the uncoated alloy.

For what concerns the disks’ aesthetic features, it has to be pointed out that the bare alloy lost its shiny aspect starting from the first minutes of treatment, while the coated ones still maintained their brightness. Proceeding with the treatment, chitosan/BTA coating maintained transparency and absence of color for a longer time than the pure chitosan film. After some hours of highly degrading aging, a slight color variation to green-yellow was observed in the case of the pure chitosan coating by visual inspection. It is worth noting that the disk’s surface coated by the pure chitosan film, as evidenced by optical microscopy images of Figure 2, was not affected by the growth of corrosion products, as the bare alloy was. The coating color variation is thus to be ascribed to an intrinsic film modification causing yellowing and homogeneity loss, clearly not occurring in the presence of the corrosion inhibitor.

### 2.3. Real Conditions Test

As previously mentioned, testing protective materials in environments as similar as possible to the real ones is of great importance for conservation studies. Accelerated degradation treatments are useful to evaluate the efficacy of new conservation materials keeping low the research duration, but they represent extreme and too aggressive conditions with respect to the ones really suffered by artifacts during storage or exhibition. Moreover, long-term effects cannot be monitored during accelerated aging treatments, but they might be significant for materials to which a long service life is required. For this reason, it was decided to carry on a second test typology with the aim of validating the coatings in a real-like indoor environment. A wooden box with fragments of plastic, leather and paper was chosen to accommodate up to 3 years the reference disks, both bare and coated with chitosan, chitosan/BTA and Incral 44 films. The context proposed is representative of the bad storage conditions suffered by artifacts in museums’ deposits or inside exhibition showcases containing different types of materials. Wood, paper, plastic, fabrics and leather are in fact materials sometimes found along with metal manufacts. They can be used as materials for the construction of deposit boxes, or they can be artistic objects themselves [32,34]. In this latter case, it is not uncommon to find artifacts of different composition, including silver ones, exhibited in the same display case. Such materials may represent a serious threat to the correct conservation of metal objects. It is well known that they naturally emit airborne degrading species, including sulfur-containing compounds, such as hydrogen sulfide (H_2_S), sulfur dioxide (SO_2_) and organo-sulfides, at concentrations able to induce degradation processes on silver surfaces [35,36]. In confined spaces such as storage rooms or showcases, where the absence of circulating air causes pollutant accumulation, even low amounts of aggressive species can promote the degradation of Ag-based alloys.

**Figure 3:**
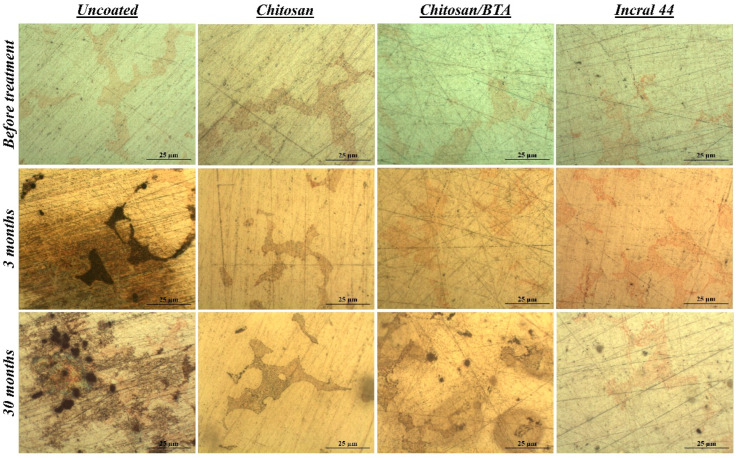
Surface features before and after the real conditions test. Optical microscopy images of a bare, a chitosan, a chitosan/BTA and an Incral 44 coated disk at different times of exposure inside the wooden box.

In Figure 3, optical microscopy images showing both the coated and uncoated surfaces, before the test and at different storage times, are reported. After only 3 months of storage, alteration products developed on the bare alloy surface, while under the chitosan-based coatings the alloy remained well preserved, confirming the protective properties already discussed in Section 2.2. But contrary to what has been previously observed, after a longer period of time the pure chitosan coating provided a better protection toward the metal substrate than the chitosan/BTA film. The surface protected by the pure chitosan did not change, while under the chitosan/BTA coating brown alterations, mainly spread in correspondence of copper dispersions, appeared. However, the chitosan-based films provided a protection comparable to that shown by the commercial reference coating.

Concerning the film’s aesthetic features, it has to be pointed out that after prolonged exposure inside the box, both the chitosan and chitosan/BTA coatings started acquiring a yellow hue, actually more pronounced in the case of the film containing benzotriazole.

### 2.4. Coatings Alteration

In order to understand the mechanisms of the coatings’ alteration, these were further analyzed by FE-SEM-EDS and UV-Vis spectroscopy. For comparison, both typologies of coatings (pure chitosan and chitosan/BTA) were also applied on glass slides and subjected to the accelerated degradation treatment with ammonium sulfide. In the case of the glass slides, no color variation was observed for the polymeric coatings, which maintained transparency for the whole treatment duration. The interaction between the film and the metal substrate was thus the one responsible for the coatings’ alteration, excluding any photochemical or environment-induced reaction as a possible cause of degradation.

**Figure 4:**
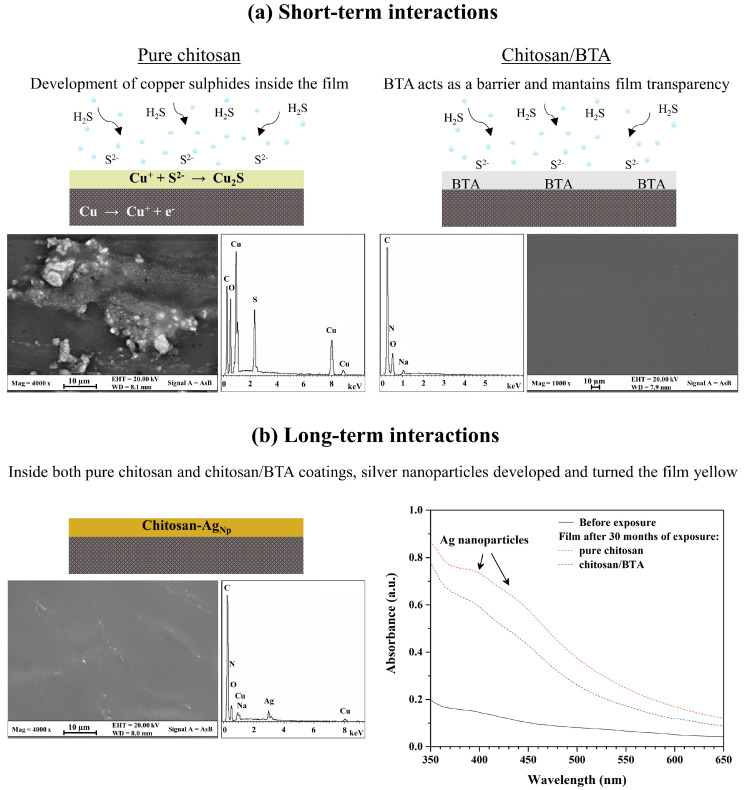
Effects of short- and long-term interactions between the chitosan-based coatings and the silver alloy. (**a**) Graphical illustration of chitosan and chitosan/BTA coatings’ appearance after 10 h of accelerated degradation treatment. FE-SEM images and relative EDS spectra show morphology and chemical composition of the coatings; (**b**) Graphical illustration of a chitosan coating representative of both pure chitosan and chitosan/BTA films’ appearance after 30 months of storage inside the wooden box. FE-SEM images and relative EDS spectra show morphology and chemical composition of the coating, while UV-Vis spectra show absorptions typical of silver nanoparticles.

The FE-SEM images and relative EDS analyses of the chitosan and chitosan/BTA coatings peeled off from the silver disks’ surface after 10 h of accelerated aging treatment are reported in Figure 4a. The film containing benzotriazole maintained homogeneity and transparency for the whole treatment duration. The aggressiveness of the corrosive environment did not affect its physico-chemical stability nor its elemental composition. In the case of the pure chitosan coating, the absence of a corrosion inhibitor passivating the metal surface allowed the alloy to be more reactive toward the aggressive environment. EDS analyses, acquired on the rounded-shape phases, revealed significant percentages of copper and sulfur, whose ratio is chemically compatible with copper (I) sulfide (Cu_2_S). Differences in the coatings’ appearance are thus related to the presence of the corrosion inhibitor. In the absence of BTA, copper migration inside the chitosan film was promoted and its reaction with sulfur to form alteration products was permitted to some extent. Once the metal species migrated into the coating, they started reacting with the sulfide ions coming from the environment and turned into powdered and colorful compounds. On the contrary, the addition of BTA to chitosan ensured the formation of stable complexes at the interface with the copper-rich areas, preventing the alloy element migration inside the film and maintaining the coating stable, colorless and transparent.

Regarding the coatings’ yellowing observed after the storage in the wooden box, it can be stated that time played a significant role in inducing film alteration. As previously mentioned, the prolonged interaction between the chitosan-based coatings and the alloy substrate (up to 30 months) led to the film yellowing, especially in the presence of BTA. The FE-SEM-EDS analyses of the pure chitosan coating after 30 months of storage, also representative of the chitosan/BTA film, are reported in Figure 4b. Differently from what has been observed for the accelerated aging test, even if significant amounts of copper are present inside the coating, corrosion compounds did not develop. However, the FE-SEM-EDS analyses disclosed the presence of nanometric particles, largely spread all over the film and highly enriched in silver. To determine if the presence of silver nanoparticles could be responsible for the yellowing observed after this type of treatment, the coatings were also analyzed by UV-Vis spectroscopy. After 30 months of exposure, both the chitosan and chitosan/BTA coatings showed absorption in the region of 400–440 nm. As frequently reported in the literature [37,38,39], nano-sized silver particles have absorption peaks in the visible region from 400 nm to 440 nm, depending on the size and morphology of the particles. These confer from yellow to light red hues to solutions or films containing them [40]. The coatings’ yellowing can thus be related to the development of silver nanoparticles inside the polymeric film.

Concerning copper and silver migration inside the chitosan-based coatings, it should be noted that chitosan’s chelating properties toward metal ions are well documented in the literature [23,41]. Its alcoholic and amine functional groups provide the electrons to which copper or silver cations can chemically or physically bind, thus forming coordination polymer-metal bonds [40]. These are favored at neutral or slightly acidic pH values [23,42], as those typical of the chitosan coatings. The tendency to form such interactions is reported to be higher for copper cations than for other ions [24]. In our experiments, significant percentages of copper were found in the pure chitosan coating after the accelerated aging treatment and in both chitosan and chitosan/BTA coatings after prolonged exposure in the wooden box. The ability of copper to migrate and diffuse inside the chitosan-based coatings is thus confirmed. But while copper migration inside the protective film was promoted even from a few hours of aggressive treatment, silver migration, diffusion and nucleation were favored only for longer periods of aging. Time was also found to be a crucial factor in determining the effects of BTA addition on silver surfaces’ passivation. According to the experiments’ results, in the short-term the presence of benzotriazole had a positive effect in the protection of the metal surface. The high affinity toward copper dispersions, which at this stage represented the most reactive site of the whole alloy, protected the element from migration and reaction with sulfur inside the coating. In the long-term, the benzotriazole’s negative effects on silver surfaces became dominant. It is likely that the metal/inhibitor bonds weakened or that the Cu-BTA and especially the Ag-BTA complexes turned soluble, leading to the migration of both the elements inside the overlying film. As suggested by absorption peaks in the UV-Vis spectra that are more pronounced for the chitosan/BTA coating compared to the pure chitosan film (Figure 4b), the formation of Ag nanoparticles was favored and enhanced by the presence of benzotriazole inside the coating. This is not surprising because BTA is reported to be a very good stabilizing agent for copper alloys, while its efficacy toward silver-based substrates seems to be unsatisfactory. In a recent work, Ramani and Meletis stated in fact that the application of BTA on pure Ag and on a Cu-60Ag alloy promotes the silver surfaces’ activation, resulting in low inhibition efficiency [25]. Relying on XPS measurements, they proposed that the reason for this behavior can probably be attributed to a weak adsorption of BTA on the silver surface or to a not advantageous arrangement of the organometallic complexes. Their findings are in good agreement with those observed after long-term aging of Ag-disks coated by chitosan/BTA.

## 3. Materials and Methods

### 3.1. Preparation of Chitosan-Based Coatings

Before using, chitosan powder (medium molecular weight equal to 190,000–310,000 Da, viscosity 200–800 cP, 75–85% deacetylated) was washed for 1 h in boiling distilled water, filtered and dried under vacuum for 12 h at room temperature. A 0.1 M water solution of D-(+)-gluconic δ-lactone (DGL) was prepared and chitosan powder was added to obtain a chitosan/water concentration of 1 wt/vol%. The dispersion was magnetically stirred for 24 h at 30 °C to achieve the total dissolution of chitosan. Then the pH was raised to approximately 5.5 by gradually adding small amounts of a 0.5 M water solution of NaOH. Finally, ethanol was added to obtain a water/ethanol ratio of 50/50 *vol*%/*vol*%. During such operations, the solution was constantly kept under magnetic stirring. To prepare the formulation containing the corrosion inhibitor with a concentration of 2% by weight with respect to chitosan, BTA was first dissolved in ethanol and then added to the chitosan solution. The chitosan and chitosan/BTA solutions were applied on the surface of reference substrates and let dry for 12 h at room temperature. For disks with an area of about 2.5 cm^2^, 125 µL of solution were applied on the metal surface to obtain about 2 µm thick films [20]. The same amounts were also applied on the surface of glass slides for comparison purposes.

### 3.2. Preparation of Commercial Reference Coatings

Incral 44, based on acrylic resin and provided by C.T.S. S.r.l. (Altavilla Vicentina (VI), Italy), was selected as commercial reference product. The original resin concentration, declared to be of 15%, was lowered to 0.5 % by dissolution in toluene to obtain a final concentration comparable to that of the chitosan-based formulations. In all, 125 µL of solution were applied on the metal surface and let dry for 12 h at room temperature.

### 3.3. Preparation of Ag-Based Reference Substrates

An Ag-based reference alloy was purposely produced for the experiment (labeled CNR 180). A chemical composition typical of sterling silver (Ag 92.5% and Cu 7.5%, by weight) was selected and a microstructure of as-cast alloys was reproduced [22]. Pure granulated Ag and Cu were mixed in the right proportions and melted in a graphite crucible placed in an electrically heated furnace with a reducing atmosphere. The rapid cooling at room temperature led to the formation of ingots with a 1.8 cm diameter. These were cut to obtain small disks, which were successively polished with SiC papers at 1200 grit and diamond pastes up to 1/4 μm.

### 3.4. Aging Tests

Two different typologies of aging tests were developed, based on short-term and long-term treatments.

The accelerated corrosion test involved the use of sulfide vapors as aggressive chemical species, able to induce silver tarnishing in a short time. The reference disks, both coated by chitosan, chitosan/BTA, and Incral 44 films and uncoated for comparison purposes, were put into a closed glass vessel containing a 1 mM water solution of ammonium sulfide ((NH_4_)_2_S). The tests were carried out at 30 °C for different time intervals. Along with the reference disks, glass slides coated by the same chitosan-based films were put in the vessel and subjected to the accelerated aging.

The real-like aging test was carried out by storing the reference substrates, both coated and uncoated, in a wooden box containing fragments of paper, leather and plastic. The test lasted for 30 months, and monitoring examinations were planned at different time intervals.

### 3.5. Characterization

To evaluate the coatings’ protective efficacy, the surface appearance of the coated reference substrates was visually compared to one of the uncoated disks, before and after the aging tests. Optical microscopy investigations were performed by using a Leica MZFLIII microscope equipped with a digital camera (Leica DFC 320) and a Leica MEF IV optical microscope equipped with a 420 Leica digital camera.

Morphological and microchemical analyses of reference substrates after the removability test and of coatings after the aging treatments were carried out by a high-brilliance and high-spatial-resolution LEO Gemini 1530 (Zeiss, Jena, Germany) field emission scanning electron microscope, coupled by an energy dispersive X-ray spectrometer INCA 450 and four-sector back-scattered electron detectors. Images were all recorded in the back-scattered mode at different acceleration voltages from 1 to 20 kV. Concerning the coatings’ investigations, to make sure that only the film’s portion was analyzed without interferences with the underlying alloy, these were peeled off from the disks’ surface by a proper adhesive tape and then put into the microscope. No sample metallization was performed before the investigation.

The removability of chitosan-based coatings was studied by Fourier Transform Infrared spectroscopy using an iS50 spectrometer (Thermo Fisher, Rodano (MI), Italy) equipped with an ATR accessory. The measurements were recorded using a diamond crystal cell and 32 scans at a resolution of 4 cm^−1^. No ATR correction was applied to the data.

Coatings’ transparency and absence of color, as well as yellowing after aging treatments, were studied by a double beam spectrophotometer V-660 (Jasco, Cremella (LC), Italy). UV-Vis measurements were performed in transmittance mode on coatings applied on glass slides, using air as a reference. The color changes after the treatments were investigated in reflectance mode by using a 60 mm integrating sphere and BaSO_4_ as standard diffuse reflectance material.

Corrosion products, grown on the uncoated reference disk after 36 h of accelerated aging treatment at high concentration of ammonium sulfide, were characterized by means of an X-ray diffraction analysis on the powder scratched from the surface. The instrument was a Siemens 5000 X-ray powder diffractometer with Ni-filtered Cu K_α_ radiation (λ = 1.5405 nm). Angular values between 10° and 65° in the additive mode, a step size of 0.04°, and a sampling time of 20 s were used as experimental parameters. The X-ray diffraction pattern was analyzed by electronic databases.

## 4. Conclusions

Two typologies of chitosan-based protective coatings were formulated, applied on reference sterling silver substrates and compared to a commercial protective coating. A pure chitosan coating was prepared with the aim of studying the polymer protection efficacy and the interactions with silver surfaces, while BTA was added to the second formulation in order to enhance the corrosion inhibition properties. UV-Vis spectroscopy and FTIR analyses confirmed the coatings are colorless, transparent and easily removable with a gauze soaked in water or ethanol.

To validate the protective properties toward silver surfaces, two different aging treatments were developed. A bare reference disk, a chitosan, a chitosan/BTA and an Incral 44 coated disks were exposed to a 1 mM water solution of ammonium sulfide ((NH_4_)_2_S) in a closed vessel for different time intervals. At the end of the test, the BTA-containing coating remained colorless and transparent, protecting the underlying alloy for up to ten hours and being totally comparable to the commercial reference one. By contrast, the pure chitosan film slightly acquired green-yellow hues due to the internal growth of copper and sulfur compounds, as demonstrated by the FE-SEM-EDS analyses. To evaluate the coatings’ properties in the long-term, a bare disk, a chitosan, a chitosan/BTA and an Incral 44 coated disks were put into a wooden box containing fragments of paper, leather and plastic and simulating the museums’ storage or exhibition degrading conditions. This time, the pure chitosan coating totally preserved the silver surface from alteration, showing great protective properties such as those of the commercial reference. Concerning the coatings’ appearance, after a long exposure (30 months), the films, especially those containing benzotriazole, started to yellow. The color change was related to the migration and nucleation of the silver nanoparticles inside the coatings, as revealed by the FE-SEM-EDS analyses and UV-Vis spectroscopy. These results suggest that in a real environment, the pure chitosan coating can perform better than that containing benzotriazole, thus opening the way to innovative, totally biocompatible protective materials.

The chitosan-based coatings developed in this study showed good and promising protective properties toward sterling silver alloy. Similar to commercial acrylic resin, they turned out to be effective in passivating and preserving the metal surfaces from alteration processes, induced by both a highly aggressive environment and a prolonged exposure to bad storage conditions. The experiments demonstrated that, despite the common use of benzotriazole for the conservation of silver artifacts, in the long-term it was not able to satisfactorily protect the Ag surfaces. Thus, the choice of a proper inhibitor that can limit the solubilization and migration of metal ions inside the polymer matrix proved to be necessary to avoid any yellowing of the coating.

Finally, the experiments pointed out that, when new protective strategies are going to be developed, both accelerated corrosion treatments and long-term tests are essential to validate the protective efficacy and to fully comprehend the interactions between the coatings and the underlying substrates.

## Data Availability

Any data or material that support the findings of this study can be made available by the corresponding author upon request.

## Supplementary Materials

The following supporting information can be downloaded at: https://www.mdpi.com/article/10.3390/ijms232214454/s1.
