# Supplementary material for: Toward a Green and Sustainable Silver Conservation: Development and Validation of Chitosan-Based Protective Coatings
Source: Int J Mol Sci. 2022 Nov 21;23(22):14454. doi: 10.3390/ijms232214454 (PMC9697002; doi:10.3390/ijms232214454)
Supplement: Supplementary file 1 [file ijms-23-14454-s001.zip › ijms-2012002-supplementary.pdf]

Supporting information

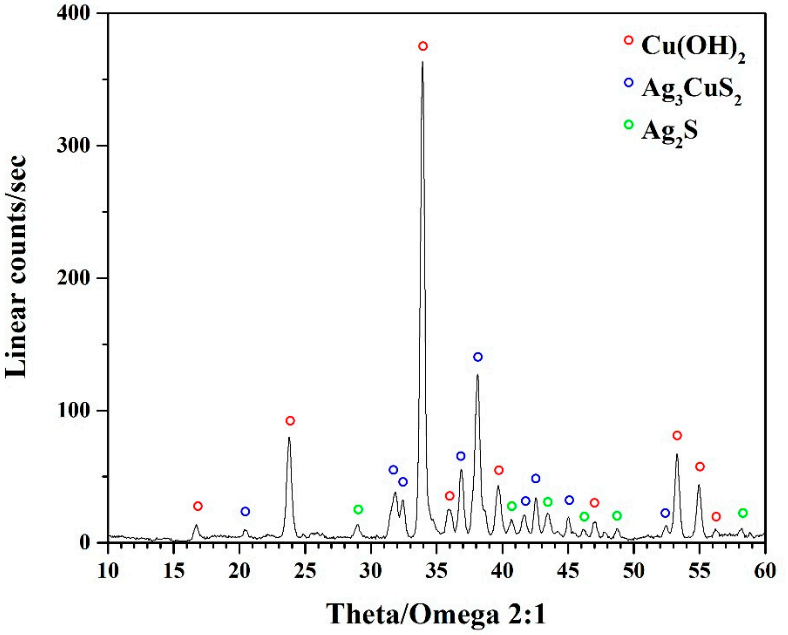

Figure S1. Powder diffractogram of corrosion products grown on a bare sterling silver disk after 36 hours of exposure to highly concentrated ammonium sulfide. The diffractogram reveals the presence of copper hydroxides ($Cu(OH)_2$), jalpaite (Ag_3CuS_2) and acanthite (Ag_2S).

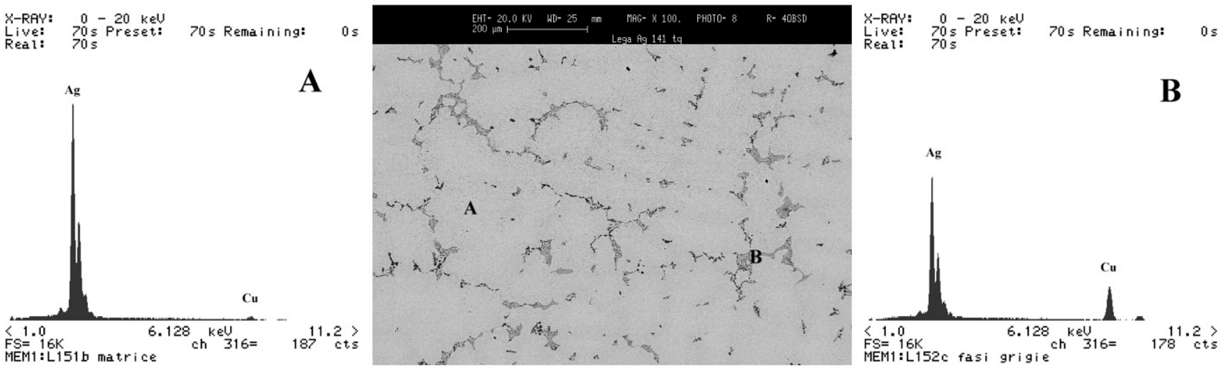

Figure S2. Micro-structure and micro-chemical composition of the reference alloy. The reference alloy used in this study was produced with a composition and a micro-structure as similar as possible to the ones commonly observed in real silver artifacts. The rapid cooling, resulting from melting and subsequent casting operations, led to a dendritic microstructure, often showing chemical segregation phenomena among the dendrites and copper dispersions occupying interdendritic spaces. The two typical phases of an "as-cast" alloy with a nominal composition of Cu 7.5% and Ag 92.5% are a copper-enriched α-phase and a surrounding silver-enriched β-phase constituting the dendrites. The presence of copper dispersions inside the silver matrix strongly affects the corrosion mechanism of sterling silver because they represent the sites where the metal oxidation begins. The connection between Ag-rich and Cu-rich areas leads indeed to the establishment of galvanic cells where the nobler element (i.e., silver), acts as a cathode and the less noble (i.e., copper), acts as an anode and preferentially corrodes. Starting from the α-phase, the process readily depletes copper and expands, affecting the surrounding β-phase.
The SEM image shows the microstructure of the reference alloy, while the EDS spectra highlight the chemical composition of two different phases, the silver rich β-phase (spectrum A) and the copper rich α-phase (spectrum B).

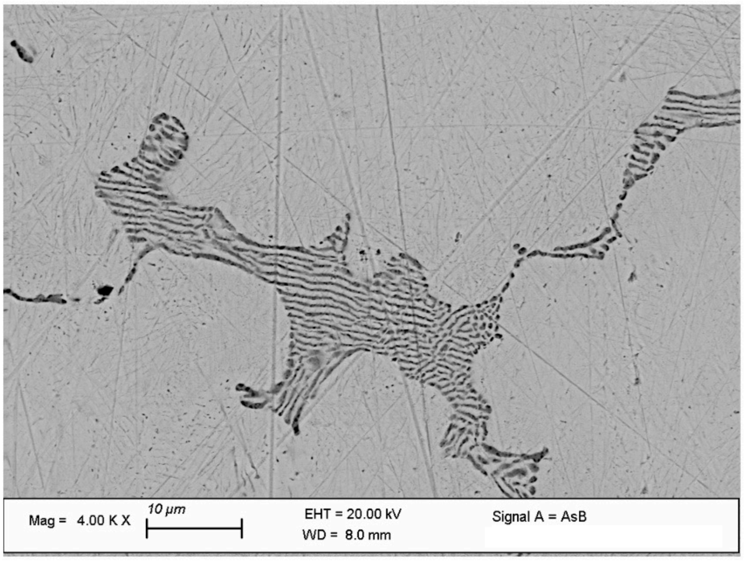

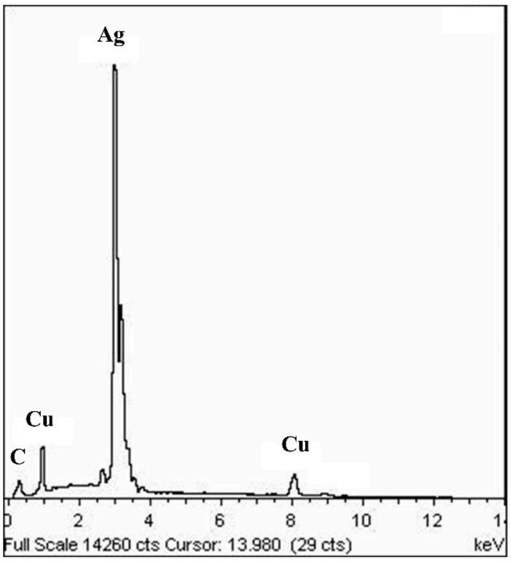

Figure S3. Sterling silver surface after removal of the chitosan/BTA coating exposed to 10 hours of accelerated aging with ammonium sulfide 1mM. The FE-SEM image and the relative EDS analysis acquired on the entire area reveal that no corrosion products developed on the surface after the aging and no coatings residues remained after the film removal.
